# Repurposing Drugs in Oncology (ReDO)—nitroglycerin as an anti-cancer agent

**DOI:** 10.3332/ecancer.2015.568

**Published:** 2015-08-27

**Authors:** Vidula Sukhatme, Gauthier Bouche, Lydie Meheus, Vikas P Sukhatme, Pan Pantziarka

**Affiliations:** 1 GlobalCures, Inc, Newton MA 02459, USA; 2 Anticancer Fund, Brussels, 1853 Strombeek-Bever, Belgium; 3 Beth Israel Deaconess Medical Center and Harvard Medical School, Boston, MA 02215, USA; 4 The George Pantziarka TP53 Trust, London KT1 2JP, UK; § Corresponding authors; * Lead authors

**Keywords:** ReDO project, drug repurposing, nitroglycerin, hypoxia, EPR effect

## Abstract

Nitroglycerin (NTG), a drug that has been in clinical use for more than a century, has a range of actions which make it of particular interest in an oncological setting. It is generally accepted that the main mechanism of action of NTG is via the production of nitric oxide (NO), which improves cardiac oxygenation via multiple mechanisms including improved blood flow (vasodilation), decreased platelet aggregation, increased erythrocyte O2 release and decreased mitochondrial utilization of oxygen. Its vasoactive properties mean that it has the potential to exploit more fully the enhanced permeability and retention effect in delivering anti-cancer drugs to tumour tissues. Moreover NTG can reduce HIF-1α levels in hypoxic tumour tissues and this may have anti-angiogenic, pro-apoptotic and anti-efflux effects. Additionally NTG may enhance anti-tumour immunity. Pre-clinical and clinical data on these anti-cancer properties of NTG are summarised and discussed. While there is evidence of a positive action as a monotherapy in prostate cancer, there are mixed results in NSCLC where initially positive results have yet to be fully replicated. Based on the evidence presented, a case is made that further exploration of the clinical benefits that may accrue to cancer patients is warranted. Additionally, it is proposed that NTG may synergise with a number of other drugs, including other repurposed drugs, and these are discussed in the supplementary material appended to this paper.

## Current usage

### Introduction

Nitroglycerin (NTG), also called glyceryl trinitrate, is an organic nitrate with potent vasodilatory effects. It has a long history of use as a coronary vasodilator, most commonly used for prophylaxis and treatment of angina pectoris, but also used as a treatment for hypertension, congestive heart failure and for the induction of surgical hypotension. As with other medical organic nitrates, such as isosorbide dinitrate and isosorbide mononitrate, NTG acts as a nitric oxide (NO) donor, which is a key vasodilatory effector molecule. NTG is a prescription drug available globally as a generic.

### Dosage

NTG is available in sublingual tablet and spray form for oral use, as a transdermal patch, as an ointment and as an intravenous infusion. For the prevention or treatment of angina attacks sublingual tablets are used at a dose of 0.3–1.0 mg, the dose repeated as required. The typical metered dose for sprays is 0.4 mg, with one or two doses sprayed under the tongue. Transdermal patches are used for longer term prophylaxis of angina, with typical dosages being 5 mg/day–15 mg/day. The ointment form of NTG is used for the treatment of anal fissure as well as prophylaxis of angina. For the treatment of anal fissure a 0.4% w/w ointment is used, whereas for angina the ointment is 2% w/w, giving a dose of approximately 0.8 mg per hour per inch of ointment.

Sublingual treatment, using either the spray or tablets, can provide rapid relief of angina symptoms, though the effect may only last 20–30 minutes. For longer term prophylaxis using transdermal patches or ointment, patients can develop tolerance, often after around two weeks of use. However, lowering the blood-nitrate concentration for extended periods can reverse tolerance. Therefore patients are advised to remove patches over-night or else to ensure they have 4 to 8 hours without patches or ointment in every 24 hours.

### Toxicity

NTG has low systemic toxicity. Common side effects include headache, dizziness, postural hypotension and tachycardia. Less common effects include nausea, vomiting, heartburn, flushing, and rash. NTG is contraindicated for those with known hypersensitivity to nitrates, severe anaemia, increased intracranial pressure or some hypotensive conditions. Caution is also advised for patients being treated with PDE-5 inhibitors (e.g. sildenafil citrate, tadalafil, vardenafil hydrochloride). These drugs have been shown to potentiate the hypotensive effects of organic nitrates, including NTG.

### Pharmacokinetics

NTG undergoes extensive hepatic and non-hepatic clearance with peak plasma concentrations achieved within 3–5 minutes for sublingual tablets or sprays, reaching 1.40 ng/ml (6.2 nM) to 1.97 ng/ml (8.7 nM) after 0.5 mg sublingual tablet and 3.96 ng/ml (17.4 nM) after 0.8 mg sublingual spray [1, 2]. For transdermal delivery, peak levels are achieved in around 60 minutes. For a transdermal system delivery at a dose of 10 mg/day, steady state levels achieved were 0.15 ± 0.12 ng/ml (0.7 ± 0.5 nM) [3, 4]. Note that all forms of NTG delivery are prone to high-levels of inter and intra-patient variability [3]. The two main metabolites of NTG are 1,2 and 1,3-glyceryl dinitrate (1,2-GDN and 1,3-GDN), which achieve higher plasma concentrations than the parent compound but which have lower vasodilatory activity; nevertheless these metabolites contribute to the pharmacological effects of NTG administration [5].

## Pre-clinical evidence in cancer—*in vitro* and *in vivo*

NO is a ubiquitous signalling, regulatory and effector molecule with diverse effects in the human body, including the vascular, neuronal and immune systems. With respect to cancer, NO is known to have dichotomous effects, both pro- and anti-tumour, depending on concentration, microenvironment and cell type [6–8]. In particular, low concentrations of NO (<100 nM) are associated with increases in angiogenesis, proliferation and resistance to apoptosis, whereas high NO concentrations (>500 nM) are associated with increased cytotoxicity and apoptosis [6]. While this biphasic property of NO is complex, it has not inhibited exploration of its use as an anti-cancer agent.

Interest in the potential use of NTG in cancer treatment arose primarily from investigations into the increased vascular permeability exhibited by solid tumours. This phenomenon, possibly related to inflammatory responses to injury or infection [9], was seen by some researchers as a potential mechanism for the targeted delivery of chemotherapeutic agents to solid tumours [10, 11]. However, subsequent investigations of anticancer effects have not been limited to vascular permeability alone.

Matthews *et al* investigated the relationship between hypoxia, endogenous NO production and the chemosensitivity of human and murine cancer cell lines [12]. Human breast cancer (MDA-MB-231) and mouse melanoma (B16F10) cells were exposed to differing levels of O2 and then incubated with an inhibitor of NO production and treated with either doxorubicin or 5-fluorouracil. Results showed that hypoxia and inhibition of endogenous NO production rapidly induced resistance to both chemotherapeutic agents. The increase in cell survival on exposure to doxorubicin was mimicked under normoxic conditions by use of an inhibitor of endogenous NO synthase, suggesting that low NO levels contribute to hypoxia-related chemoresistance. This effect could be partially reversed using low doses of NTG (1 μM and 0.1 nM) or diethylenetriamine NO adduct (1 μM).

Hypoxia is a known driver of increased tumour invasiveness and the effect of NTG on this process was assessed by the same group, also using the MDA-MB-231 breast cancer cell line [13]. Hypoxic conditions increased Matrigel invasion five-fold compared to normoxia, an effect abolished by the use of NTG (again at the low dose of 1 μM and 0.1 nM) or sodium nitroprusside. Similarly, in vivo investigations on the effect of hypoxia on metastases in a melanoma model showed that NTG reversed the increase in metastatic nodules induced by hypoxic conditions [14].

Other in vitro investigations of hypoxia, chemosensitivity and NTG have included prostate cancer [15, 16] and breast cancer three dimensional tumour spheroids [17].

Escape from immunosurveillance may also be a consequence of tumour hypoxia, and this was investigated by Siemens *et al* in prostate cancer cell lines and a murine xenograft model (using the human PC-3 prostate cancer cell line) [18]. The study showed that impaired NO signalling, associated with hypoxia, increased tumour cell shedding of MHC class I chain-related (MIC) molecules and thus contributed to immune escape. This effect could be reversed with exogenous NO using NTG, and in the murine model transdermal patches, delivering NTG at a rate of 7.3 μg/h, were used to attenuate the growth of xenografted MIC-expressing human prostate tumours compared to placebo. Later studies further elucidated this effect using the human prostate cancer DU145 cell line implanted into athymic nu/nu mice treated with placebo or NTG patches and showed a similar reduction in the rate of tumour growth [19].

Maeda and colleagues first proposed the term ‘enhanced permeability and retention effect’ (EPR Effect) in a Japanese paper in 1987 to refer to the intratumoral accumulation of large molecules due to the increased vascular permeability of tumours and the lack of lymphatic drainage [20]. Modulation of these vascular features for therapeutic advantage have been investigated in a number of in vivo models and using a variety of agents by Maeda and co-workers starting in 1998 [21–23]. In vivo manipulation of the EPR effect using NTG in both rat (breast cancer) and mouse (S-180 sarcoma, fibrosarcoma and colon adenocarcinoma) models of cancer were investigated by Maeda and colleagues, who used topical application of NTG ointment to show an increased accumulation of anticancer drugs and other macromolecules [24]. Of note was that the increased accumulation of agents was shown to be consistent in different tumour types, anatomical sites and macromolecular agent.

The use of NTG in lung cancer has also been investigated in vivo, in particular in combination with the anti-folate chemotherapeutic drug pemetrexed [25]. Murine Lewis Lung Carcinoma cells were implanted into NCr C57/BL6 mice, and following tumour development were treated with either saline, NTG, pemetrexed, NTG + pemetrexed or NTG + pemetrexed or 1H-[1, 2, 4] oxadiazolo [4,3-a]quinoxalin-1-one (ODQ). Treatment with the combination of NTG and pemetrexed showed the greatest reduction in tumour growth, (tumour volume was approximately 20% of untreated control at day 15 after start of treatment, P < 0.05), an effect that was reversed by the addition of ODQ.

## Human data

While the pre-clinical evidence is suggestive of an anti-cancer effect of NTG, in general in drug repurposing it is human data which is of greater translational significance [26]. In this case there are a number of clinical studies which are in line with the pre-clinical data.

### Lung cancer

A randomised controlled Phase II trial of NTG in addition to vinorelbine and cisplatin in stage IIIB/IV inoperable non-small cell lung cancer (NSCLC) was initiated in Japan following an unpublished analysis of response rates comparing lung cancer patients with angina pectoris treated with NTG and patients with lung cancer only [27]. 120 treatment-naïve patients were randomised to four cycles of vinorelbine and cisplatin and placebo patch or vinorelbine and cisplatin with a transdermal NTG patch (25 mg/patient daily for five days per cycle, starting three days prior to chemotherapy). Primary end-points were response rate and time to disease progression. The response rate in the NTG arm was 72%, which was significantly (P < 0.001) higher than the 43% for patients in the placebo arm. The median time to progression was 327 days for the NTG arm, compared to 185 days for the placebo arm (HR = 2.1; 95% CI 1.3–3.2; P = 0.002). Median overall survival was 413 days (range 32–1380 days) in NTG, and 289 days (range 56–1117 days) in placebo arm, (HR = 2.5; 95% CI 1.6–3.9; P < 0.001).

Subsequent work by the same group examined the combination of NTG and docetaxel and carboplatin and the impact of chemosensitivity in two small cohorts of operable lung cancer patients [28]. In the first group, 17 patients with lung cancer and stable angina pectoris (of whom eight had been treated for more than 3 months with transdermal NTG patches delivering 25 mg/day), had their resected tumours immunohistochemically analysed to investigate the long-term effects of NTG treatment on HIF-1α, P-gp, VEGF, p53, and activated p53 (phosphorylated p53 at serine 15). Results showed that staining for HIF-1α, P-gp and VEGF was significantly lower in NTG treated patients compared to controls, although there was no difference between groups for p53. In the second cohort, 29 patients with advanced NSCLC were treated with transdermal NTG (at a dose of 25 mg/day) with each cycle of chemotherapy with docetaxel and carboplatin. The five-day treatment commenced three days prior to chemotherapy. Treatment with NTG resulted in lower plasma VEGF levels compared to baseline, which correlated with sensitivity to chemotherapy response. The response rate in these patients was 59%, which compares well to the typical response of 24–36% to this chemotherapy regimen in similar populations.

An on-going trial by the same group is investigating the use of NTG with paclitaxel and carboplatin in treatment-naïve Stage III/IV NSCLC patients, (NCT00616031 – current status of trial unknown according to ClinicalTrials.gov as at June 2015).

There are also Japanese case reports of the use of NTG with the anthracycline amrubicin in five heavily pre-treated patients with advanced NSCLC [29]. All five patients, suffering refractory and recurrent disease, had been treated with at least three prior lines of chemotherapy before being treated with amrubicin and transdermal NTG. Two of the five patients achieved partial response; one patient had received three prior regimens and the other four. Both patients experienced partial response with transdermal NTG at a dose of 10 mg/day.

The Japanese trial of NTG with vinorelbine plus cisplatin was replicated in a Phase II trial in Germany, using a similar protocol and in a similar patient population, although in this case oral vinorelbine was used [30]. Treatment-naïve patients with stage IIIB/IV NSCLC were randomised to oral vinorelbine and cisplatin with NTG or placebo patch. The NTG dose was 25 mg/day. The trial was terminated early due to lack of recruitment, with only 66 patients treated. While the results did not reach statistical significance, the overall response rate (35.3% vs. 18.8%) and the disease control rates (61.8% vs. 53.1%) were higher in the NTG arm than in the placebo arm, although there was no difference in progression free survival (PFS) or overall survival (OS). The discordance between the overall response rate and the lack of difference in PFS and OS measures is suggestive of predominantly transient responses induced by NTG.

Negative results were reported for the NVALT12 Phase II randomised open-label trial of paclitaxel, carboplatin and bevacizumab (PCB), with and without transdermal NTG in patients with stage IV non-squamous NSCLC. Patients randomised to the NTG arm (n = 111) were treated with PCB and NTG patches delivering 15 mg/day for 5 days (starting two days prior to each of four 3-week chemo cycles), the control arm (n = 112) received the same treatment without the NTG patch. Treatment with bevacizumab or bevacizumab + NTG continued as maintenance therapy until disease progression [31]. The response rate was 30% for the NTG arm, compared to 45% for control, median PFS 5.0 months (4.2–5.8) for NTG and 6.8 months (5.6–7.3) for the control, with HR of 1.22 (95% CI 0.91–1.63). Median OS was 9.5 months (7.8–11.9) for NTG and 11.6 months (8.7–14) in control, with HR of 1.12 (95% CI 0.76–1.67).

Another negative trial has also reported results in NSCLC. The Australasian NITRO study was a large phase III trial of NTG in addition to first line chemotherapy in advanced NSCLC (stage III and IV). Results reported at ESMO 2014 showed that the trial was terminated early after the first interim analysis for no demonstrable advantage in terms of PFS with the addition of NTG. Patients in the NTG arm were treated with NTG patches (delivering 25 mg/day) in addition to a variety of first line regimens (mainly carboplatin with either paclitaxel or gemcitabine). Objective tumour responses were seen in 26% of NTG patients compared to 31% in the non-NTG patients, and stable disease was recorded in 49% in the NTG group versus 45% in the non-NTG group. Median overall survival was 10.8 months with NTG and 11.2 months without [32]. A meta-analysis, (excluding the data for cisplatin and vinorelbine), presented at the same meeting by the NITRO authors showed that the addition of NTG did not improve outcomes for NSCLC patients treated with carboplatin with either paclitaxel or gemcitabine.

In addition to looking at NTG with chemotherapy, there has also been investigation to ascertain whether NTG is effective with chemo-radiotherapy. A non-randomised Phase II trial in NSCLC of NTG, oral vinorelbine, cisplatin and concurrent radiotherapy has also reported results in Mexico [33]. Thirty-five patients were enrolled in this trial, of whom 63% achieved a clinical response after induction of chemotherapy, and 75% achieved a clinical response after chemo-radiotherapy. The computed 2-year PFS was 38.1% and median overall survival was 26.9 months, with a 2-year OS rate of 51.3% which was close to, but below, the target rate of 55% used in the design and powering of the trial, and compared to a 2-year actuarial survival rate of 30% in a similar patient population. A reduction of plasma VEGF levels was positively associated with improved OS in patients in the NTG arm, with the median OS for patients who achieved > 50% VEGF level reduction was 42.9 months (95%, CI 20.8–64.9) compared to 19.5 months (95% CI, 17.6–31.4) for patients with <50% reduction (P = 0.043).

The impact of NTG on chemo-radiotherapy is also being investigated in an on-going Phase II trial in the Netherlands (NCT01210378). Some initial results of this trial are discussed in the Our Take section of this paper.

There is some evidence to suggest that some of the clinical effects in NSCLC may be specific to NTG and not a feature of NO-donors in general. For example a trial compared two different chemotherapy regimens (irinotecan and cisplatin versus irinotecan and capecitabine), with and without the NO-donor isosorbide-5-mononitrate (ISM) [34]. While results showed that there was little difference between the two chemotherapy regimens in terms of overall survival, the addition of ISM did not show improvement in any treatment outcome (response rate, progression free survival or overall survival). The study authors speculated that the lack of efficacy may have been related to the lack of nitrate-induced mitochondrial ROS production compared to NTG, and that this may have contributed to these negative results.

### Other cancers

In prostate cancer a prospective, open-label trial of 29 men with an increasing prostate-specific antigen (PSA) level after surgery or radiotherapy investigated the use of transdermal NTG [35]. Patients were enrolled on a 24-month trial to compare PSA doubling time (PSADT) before and after treatment initiation, as well as comparison with a matched control group that received no immediate treatment for their PSA recurrence. The trial used very low doses of NTG, using commercially produced patches cut-down to a custom size, delivering a dose of 0.033 mg/h continuously. PSA levels were monitored monthly for the first 6 months and then every 3 months for the rest of the trial. Of the 29 patients in the trial, 17 completed the full 24-months of treatment, and only 3 of 29 had documented disease progression (10%) by the end of the trial. The results showed that the treatment group had a calculated PSADT of 31.8 months, compared to the pre-treatment rate of 13.3 months or that of the matched control group at 12.8 months.

NTG has also been investigated as a potential agent to improve response in liver cancer patients treated with transcatheter arterial embolization (TAE) or transcatheter arterial chemoembolization (TACE) [36]. In a single-site randomised controlled study, 101 hepatocellular carcinoma (HCC) patients with early to middle stage disease (BCLC A or B), were randomised to receive TAE/TACE with or without NTG. Patients receiving TACE were treated with the addition of doxorubicin to the lipiodol carrier, otherwise the treatment was the same as for TAE. The NTG dose of 100 μg was injected via the catheter immediately prior to the injection of lipiodol or lipiodol/doxorubicin. Results showed that the addition of low dose NTG increased the volume of lipiodol retained in tumours, and led to a greater reduction in tumour diameter. Although these results were statistically significant, the improved overall response in the NTG arm (98.1% vs 92%) was not, (the 92% response rate in the control arm limited the ability to detect a significant increase).

Finally, a small open-label Phase I dose-escalation study investigated the safety and tolerability of transdermal NTG patches in the neoadjuvant treatment of resectable rectal cancer, alongside chemo-radiotherapy with 5-FU [37]. The trial concluded that the treatment was safe at the maximum tested dose of 0.6 mg/h, and recommended this dose be used in subsequent Phase II trials. While safety and tolerability were the key end-points, the trial also reported a pathologic complete response rate of 17%, a figure within the range of current standard of care.

## Clinical trials

A number of clinical trials are currently (June 2015) investigating the addition of NTG to existing cancer treatments.

Trial NCT01210378 is a Phase II study investigating the use of transdermal NTG in addition to standard of care treatment (chemoradiotherapy) for NSCLC. Specifically the study aims to track tumour hypoxia and perfusion. The primary outcome is an increase in overall survival of 15% compared to historical controls after 2 years.

Trial NCT00616031 is a Phase II trial of paclitaxel and carboplatin with or without NTG in patients with previously untreated stage III or IV NSCLC. The trial is randomised and controlled with tumour response rate as the primary outcome.

Trial JPRN-UMIN000000813 is a Phase II trial of amrubicin and NTG in third-line chemotherapy in patients with NSCLC. This is a single arm, non-randomised study with PFS as the primary end point.

Trial JPRN-UMIN000001820 is also in NSCLC. This is a phase II trial of NTG with docetaxel in elderly untreated stage IIIB/IV NSCLC. This is a single arm trial with response rate as the primary end point and PFS as a secondary outcome.

Trial NCT01704274 is Phase III study of two different low-dose applications of transdermal NTG in men with biochemical evidence of recurrence (increasing PSA levels). This randomised placebo controlled trial will track markers of immune response in addition to PSA.

## Mechanism of action

There are multiple mechanisms of action posited to explain the diverse anticancer effects of NTG. These include:

- Vasodilation leading to enhanced permeability and retention (EPR) effects
- Anti-hypoxic activity
- Immunomodulation
- Pro-apoptopic effects

### NO release/vasodilation

Although NTG has more than 100 years of clinical use as a vasodilatory agent, through its action as a NO donor, understanding of the molecular basis for this effect has remained elusive. Multiple mechanisms and pathways for the conversion of NTG to NO or NO precursors have been elucidated, including xanthine oxidase, glutathione S-transferase, mitochondrial aldehyde dehydrogenase (ALDH2) and via the phosphatidylinositol 3-kinase (PI3K) pathway. Currently it is the latter two pathways which are garnering the most interest in that they can explain the generation of NO, and consequent vasodilation, at clinically relevant nanomolar doses of NTG.

In 2002 Stamler *et al* published a paper that reported a mechanism of activation via mitochondrial ALDH (ALDH2) [38]. They showed that ALDH2 generated the right mix of metabolites (1,2-GDN in preference to 1,3-GDN), released NO, caused vasodilation and could induce nitrate tolerance. However, the in vitro concentrations used were 0.1 μM–10 μM which are high, and nitrate tolerance was induced at extremely high concentrations (0.3 mM). Later work extended the analysis using ALDH2 knockout mice using very similar dose levels [39]. Other work looked at using ALDH2 inhibitors along with relevant doses of NTG in animals and noting the reduction in vasodilatory activity. Not every step in the process of going from NTG to NO was elucidated by this work, as the authors made clear in their papers. Mackenzie *et al* also found that in humans, ALDH2 is involved in the transformation of NTG and subsequent vasodilation but that it accounts for less than half of the total bioactivation [40].

Beretta *et al* identified a possible NTG bioactivation mechanism via cytosolic ALDH (ALDH1) in a bid to elucidate some of the missing steps in the ALDH2 mechanism [41]. Highly purified ALDH1 and ALDH2 and extremely high concentrations of NTG (100 μM and 10 μM for ALDH1 and 2 respectively) were used. At these high in vitro concentrations ALDH1 showed some bioactivation of NTG, but as the authors concluded: *The low apparent GTN [NTG] affinity of ALDH1 is expected to limit the contribution of this isoform to GTN bioactivation in patients treated with therapeutically relevant low doses…* Tsou *et al* also investigated ALDH1 (specifically ALDH1A1) in vitro [42]. Again very high concentrations of NTG were used – 0.1 mM and 1.0 mM – thereby casting doubt on the relevance of this in vivo.

Subsequently, work by Bonini *et al* in 2008 proposed an alternative explanation in which they used in vitro and in vivo experiments to show that signal transduction by NTG via endothelial NOS is the active mechanism of action at low nanomolar doses [43]. This mechanism of action views NTG as a signalling molecule that activates eNOS generation of NO at very small doses (1 nM) and at very rapid timescales (< 1 minute). In the same way that the Stamler group used ALDH2 inhibitors to show that ALDH2 inhibition or knockout mice reduced NTG activity, the Bonini group used NOS inhibitors and knockout mice. Subsequent work further elucidated the signalling mechanism, implicating PI3K/PKB/PTEN [44]. In this they also accept that at higher doses (> 50 nM) metabolic effects may predominate.

Regardless of the direct mechanisms of action, and it is likely that there are multiple redundant pathways operating given the physiological importance and ubiquity of NO and NO-signalling, clinically there are a number of observations that are of significance with respect to NTG and its vasodilatory activities. The first is that the NO-generating activity of NTG is substantially increased during hypoxia, effectively acting to target the effect to those tissues with low oxygenation such as solid tumours or cardiac infarct tissue [45, 46]. Secondly, given the physical characteristics of tumour-associated vasculature – increased permeability, chaotic organisation, abnormal structure [47] – there is evidence that increasing permeability using NTG can lead to the increased retention of macromolecules in tumour tissues (the EPR effect) [46, 48, 49].

### Hypoxia

Tumour tissue is subject to both chronic and transient hypoxia related to the chaotic structure of the vasculature. Tumour hypoxia is associated with increased genomic instability and the development of more aggressive cancer phenotypes, resistance to treatment, increased metastatic potential and worse prognosis in a range of cancer types [50]. Hypoxia-inducible factor 1-alpha (HIF-1α) is the primary transcription factor involved in the cellular response to low-oxygen conditions, transcribing for pro-survival responses such as increased angiogenesis (including upregulation of VEGF), metabolic adaptation and increased proliferation [51]. Early in vitro evidence showed that NO was an inducer and stabiliser of HIF-1α in both hypoxic and normoxic inflammatory environments [52]. However, later work suggested that the effect was concentration dependent, and low NO concentrations inhibited HIF-1α stabilisation, but that at higher concentrations NO acted to stabilise it [53]. Other work has shown that that NO can suppress HIF-1α activity, and that exogenous NO-donors may act to inhibit HIF-1α expression and downstream targets [54–56].

There is clinical evidence of an effect of NTG on expression of HIF-1α, with immunostaining of tumour samples showing decreased levels of expression of HIF-1α and VEGF in NSCLC patients treated with NTG patches compared to non-treated controls [28]. Lower levels of HIF-1α were also correlated with increased response to chemotherapy with docetaxel and carboplatin. Note that this action on HIF-1α may also impart some anti-angiogenic activity following NTG treatment due to the down-stream reduction of VEGF expression.

### Immunity

Recently there has been an increased investigation of the role of HIF-1α in the process of immune escape in cancer [57]. One possible mechanism links HIF-1α to an increased expression of metalloproteinase ADAM10 leading to a decrease in surface MHC class I chain-related molecule A (MICA) levels, which in turn leads to tumour cell resistance to lysis mediated by innate immune effectors [18]. In an in vivo mouse model transdermal NTG patches attenuated tumour growth via a mechanism dependent on adaptive immune effector cells, presumed to be due to inhibition of HIF-1α accumulation and hypoxia induced PD-L1 expression [58].

It should be noted that there are concerns regarding the effects of NO on immune function. NO is a key regulatory and effector molecule with diverse effects on immunity. NO generated by iNOS is known to be involved in the regulation of lymphocyte populations, particularly T cell sub-types, and is also a key component of the lytic action of macrophages [59–61]. The action of exogenous NO on immune function is complex; for example there is some in vitro evidence for a non-specific immunosuppressive action for NTG [62], and in a rat model of atherosclerosis repeated exposure to NTG acted on macrophages to up-regulate matrix metalloproteinase-9 (MMP-9) and down-regulate tissue inhibitor of metalloproteinase-1 (TIMP-1) [63]. Additionally, there is evidence that NO is a key mediator in T-cell suppression by myeloid derived suppressor cells (MDSC) [64, 65]. These issues are further discussed later in this paper.

### Apoptosis

A final putative mechanism of action of NTG in cancer is through a direct pro-apoptotic effect. The earliest evidence for a direct cytotoxic effect of NTG was shown in a number of human leukaemia cell lines [66]. Apoptosis was associated with cytochrome c release and caspase activation in Jurkat leukemic cell lines. Subsequent work also showed an effect in human colon cancer cell lines (SW480, SW620, and HCT-116), again associated with caspase activation [67]. Analysis showed that NTG increased Fas-receptor expression and decreased levels of several endogenous inhibitors of apoptosis and thus sensitised cells to Fas-ligand-mediated apoptosis. This process has been shown to be connected to the S-nitrosylation of cysteine residues 199 and 304 in the cytoplasmic part of Fas, suggesting that treatment with NTG may restore sensitivity to Fas-L mediated tumour cell death [68].

## Our take

The pre-clinical and clinical evidence outlined in the preceding sections, (and summarised in Table 1), along with multiple putative mechanisms of action, suggest that there is a clinical role for NTG in the treatment of cancer. In particular, there is evidence that the effect of NTG in the EPR effect warrants continued investigation as a potentiating agent for existing standard of care chemo and chemo-radiation protocols. While there are some clinical trials on-going in this respect, recent negative results in NSCLC suggest that additional attention is required in the selection of agents to pair with NTG, in the timing of NTG administration within this treatment combination, potential biomarkers of response and in the characteristics of the patient population.

The most negative of the NSCLC trials was the NVALT 12 study, which has been the first to combine NTG and bevacizumab, in addition to carboplatin and paclitaxel [31]. The combination of NTG and bevacizumab, which were both administered until disease progression, may be a problematic one in that there is evidence of contrary effects of each drug on vascular permeability. Whereas NTG is well-characterised as a vasodilator able to enhance the permeability of the tumour vasculature, there is strong evidence that bevacizumab reduces permeability [69–72]. It may well be that the impaired response in the trial was due to antagonism between NTG and bevacizumab, and that the cross-talk was bi-directional with NTG interfering with the therapeutic action of bevacizumab and vice versa. In particular one must note that bevacizumab decreases chemotherapy delivery to tumour [69] while NTG increases it [46]. Additionally, bevacizumab is known to induce hypoxia and increase expression of HIF-1α [73, 74], potentially mitigating some of the anti-hypoxic effects of NTG. One may also note that the NVALT 12 study used a lower dose of NTG (15 mg/day) than the other trials in NSCLC (25 mg/day).

However, even if we exclude the NVALT 12 study due to the possible confounding with bevacizumab, there is a discrepancy between the early positive results in the Japanese trials, and the results from the non-Japanese trials that have also taken place. One possible explanation lies in the ethnic differences between patient populations. Some, (but not all [75]), studies, have suggested that polymorphisms in mitochondrial ALDH2, one of the enzymes presumed to be central in the bioactivation of NTG, have a different distribution between East Asian/Japanese populations and European populations and that these are clinically significant [40]. If this were the case it is conceivable that these differences are reflected in the divergent results in the NTG + chemotherapy trials. However, further work is required to investigate whether the ALDH2 polymorphism/ethnic differences hypothesis is correct.

In the absence of a clear explanation for the discrepancy between the Japanese and non-Japanese results we must await the results from the on-going trials to ascertain the degree to which NTG co-treatment with chemotherapy or chemo-radiotherapy benefits patients with NSCLC in terms of PFS and OS. Tracking patients for metrics related to tumour hypoxia or VEGF status may also shed more light on the biological mechanisms involved in the response. One indication that the degree of hypoxia is relevant comes from data reported by Philippe Lambin and colleagues at the 3rd ESTRO Forum in April 2015 [76]. Based on preliminary data from trial NCT01210378 and additional in vitro analysis, the results showed that in patients with hypoxic tumours NTG reduced the hypoxic fraction and volume, and that this was associated with a reduced oxygen consumption rate. The authors suggest that patient selection criteria are required to identify those with hypoxic tumours who will benefit from NTG administration.

Another concern regarding the use of NTG is its action as an NO-donor and the effect this may have on cell mediated immunity. As discussed previously, there is evidence that NO may be implicated in the immunosuppressive activity of MDSC. While NO signalling is involved, there are some indications that the effect on MDSC is indirect and mediated by tumour iNOS-associated production of VEGF, which is pivotal in the MDSC induction from myeloid precursor cells [65]. In contrast there is evidence of a positive impact of NTG on immunosurveillance of tumours in an animal model [58]. Given the potentially dichotomous roles of NTG on immune status, it is suggested that in addition to further research, clinical application of NTG with agents that have anti-MDSC action should be investigated. A number of possibilities in this direction are discussed in the supplementary information.

We must also take note that while the EPR effect is well-established in animal models, clinical results have yet to meet expectations [77]. One key criticism of a simplistic reading of the EPR effect is that it ignores the effect of the increased interstitial pressure within tumours [78–80]. While the tumour vasculature may be more permeable than normal vasculature, large molecules may be unable to cross into, and therefore accumulate, into the interior of the tumour. A strategy to address this issue is therefore required if we are to maximise the potential of the EPR effect through the use of NTG. A set of proposals are made in the supplementary materials included with this paper for drug combinations which may address this issue.

Given the evidence as it stands, it is suggested that additional clinical trials of NTG are warranted in the following cancer types, and that NTG treatment may be best suited in tumours with large hypoxic fractions to capitalize on its effects on reducing HIF-1α:

- NSCLC
- Prostate cancer
- Colorectal cancer
- Breast cancer
- Pancreatic

## Conclusion

It has long been acknowledged that treatment resistance and significant toxicity are two of the leading causes of treatment failure and subsequent mortality. The use of NTG offers an opportunity to address both of these issues. Hypoxia is associated with treatment resistance in both chemotherapy and radiotherapy; it is a driver of metastatic disease and the evolution of more aggressive tumour phenotypes. In targeting HIF-1α NTG acts as an anti-hypoxic agent, thus down-regulating the pro-survival pathways that contribute to treatment resistance. In addition by exploiting the EPR effect, NTG can help to target anti-cancer agents to disease, thus sparing host tissues and increasing the therapeutic effectiveness of a wide range of agents.

Finally, it is suggested that to maximise the potential of NTG in cancer therapy we look to expand the range of drugs with which it is combined. In doing so we can more fully address the specifics of tumour vasculature that the EPR seeks to address, and a range of proposals are made in the supplementary materials.

## Author contributions

Primary authors: Vidula Sukhatme and Pan Pantziarka. Contributing authors: Gauthier Bouche, Lydie Meheus and Vikas P. Sukhatme. All authors read and approved the final manuscript.

## Competing interests

The authors declare that they have no competing interests. All the authors are associated with not for profit organisations that aim to repurpose drugs for oncology treatments.

## Figures and Tables

**Table 1.** Summary of pre-clinical evidence by cancer type.

Cancer Type	In Vitro	In Vivo	Case Report/Trial
**Breast**	[12]		
**Prostate**	[15, 16]	[18]	[35] NCT01704274
**NSCLC**		[25]	[27–30, 33, 76] NCT01210378
**Melanoma**	[12]		
**Colorectal**	[67]		[37]

**Table 1.** Proposed drug combinations with NTG and standard of care in different cancers.

Disease	Targets	Drug combination
**Breast cancer**	Increase chemo/radio-sensitivity, improve EPR effect, target tumour-associated macrophages	Aspirin [49] Zoledronic acid [10]
**Prostate**	Increase chemo/radio-sensitivity, improve EPR effect, increase cytotoxicity, microtubule disruption	Statins (*NCT01992042*) Mebendazole [50] Metformin (*NCT01561482*)
**NSCLC**	Increase chemo/radio-sensitivity, improve EPR effect, AMPK/mTOR, COX-2 inhibition and immunomodulation	Metformin (*NCT01997775*) Diclofenac or Celecoxib (*NCT00520845*)
**Melanoma**	Microtubule disruption, anti-angiogenic and immunomodulation	Diclofenac or Celecoxib [51] Mebendazole [52] Cimetidine [53]
**Colorectal**	Microtubule disruption, AMPK/mTOR, immunomodulation, anti-histamine, COX-2	Cimetidine [36] Mebendazole [54] Metformin (*NCT01941953*) Aspirin [55]

Note that references to clinical trials or published papers are indicative of trials or case reports where the drug (or analogue) has been used for the specific indication.

## Appendix 1. Repurposing Drugs in Oncology (ReDO)—nitroglycerin as an anti-cancer agent—supplementary material

### Introduction

The following drugs warrant further investigation in combination with nitroglycerin (NTG) *and* existing standard of care chemotherapy and radiotherapy protocols, both in pre-clinical studies and potentially in clinical trials. These combinations, listed in Table 1, have been selected on the basis of existing pre-clinical and clinical experience in each of the indications. In some cases these combinations replicate existing protocols currently being tested in clinical trials, but substitute known and repurposed drugs for the newer and/or more toxic agents currently being investigated. All these proposed combinations are expected to display relatively low toxicity and use low cost and generally available agents. *The following drugs are not listed in order of priority.*

### EPR

A number of existing non-cancer drugs with evidence of anticancer activity may benefit from the enhanced permeability and retention effects which NTG can induce. A selection is listed below.

- Mebendazole – The anti-helminthic benzimidazole mebendazole (MBZ) has strong pre-clinical evidence of anti-cancer effects with a multiple possible mechanisms of action and is currently being assessed in a number of clinical trials as an anti-cancer agent [1]. MBZ is known as a microtubule disrupting agent, though with lower toxicity than some of the more commonly used microtubule disrupters of the taxane or vinca alkaloid classes. One of the obstacles to tumour retention of anti-cancer drugs, even in the face of increased vessel permeability due to NTG administration, is the increased interstitial pressure in solid tumours [2]. There is some evidence that microtubule disrupting agents from the taxane class can reduce this pressure, thereby facilitating the increased absorption of anti-cancer agents [3]. The combination of NTG and MBZ may therefore be a synergistic combination to improve the EPR effect with existing standard of care treatments.
- Zoledronic Acid – Nitrogen-containing bisphosphonates, (zoledronate, ibandronate, pamidronate etc), have a long history of clinical use in cancer to prevent and treat skeletal-related events in multiple myeloma and bone metastases [4, 5]. This activity has been related directly to action on bone resorption, for which bisphosphonates are primarily used in the context of treating osteoporosis and other bone-related conditions. However, there has also been an increasing interest in bisphosphonates for their non-bone-resorptive effects in cancer, particularly with respect to effects on the immune system [6–8]. There is also clinical evidence of a positive effect when bisphosphonates are used as adjuvant therapy in breast cancer [9]. It has recently been shown that the extra-skeletal anticancer effects of zoledronic acid may be due to targeting of tumour-associated macrophages, and that zoledronic acid accumulates in tumour tissue due to leaky vasculature [10]. Using NTG to further enhance drug accumulation warrants investigation, particularly in breast cancer.
- Macromolecules – Maeda and colleagues propose the use of macro-molecular agents (>40 kDa) to fully exploit the EPR effect with NTG [11, 12]. Examples include anaerobic bacteria (for example *Lactobacillus casei*) [13], gold nanoparticles [14], paclitaxel albumin-bound nanoparticles (Abraxane) and other large molecular agents.

### Anti-hypoxic

Hypoxia is implicated in resistance to both chemotherapy and radiotherapy in a wide range of cancer types. In this respect NTG has shown some evidence of positive effect and it is proposed that this be further investigated.

- Hormonal therapy for prostate cancer – A Phase II study has shown that very low dose transdermal NTG can slow PSA doubling time for men with relapsed disease [15]. Given these strong results, presumed to have been associated with a reduction in tumour hypoxia, further work is justified. Firstly the results should be replicated in a larger and fully randomised Phase III trial. Secondly, investigation of the effect of NTG on patients with hormone resistant disease is also warranted, particularly in light of the lack of toxicity and low costs associated with this treatment.
- Radiotherapy – There is some evidence that aberrant tumour vasculature and hypoxia are key drivers of resistance to the therapeutic effects of radiotherapy [16, 17]. Therefore addressing this issue of radioresistance is an important strategy in a number of cancers where radiotherapy is used primarily with curative intent, including head and neck cancers, cervical cancer, rectal cancer, oesophageal and gastric cancer. There is some evidence that NTG can improve radiosensitivity in NSCLC, possibly by its anti-hypoxic activity [18]. While this combination continues to be explored in NSCLC in trial NCT01210378, the mechanism of action appears not to be specific to lung cancer and therefore a rationale exists to explore the combination in other cancers.
- Celecoxib/NSAIDs – A number of different NSAIDs are being actively investigated clinically in cancer due to diverse positive effects in terms of anti-inflammatory, anti-angiogenic and possibly anti-hypoxic activity. For example there is pre-clinical evidence that the NSAID indomethacin and COX-2 inhibitor NS-398 exert an anti-hypoxic effect, in part by down-regulating expression of HIF1-α [19]. A more clinically relevant COX-2 inhibitor is celecoxib, which also shows evidence of anti-hypoxic activity [20, 21]. Celecoxib has shown positive results in a clinical trials in a number of different cancer types combined with standard of care treatments, including prostate [22], colorectal [23], and heavily pre-treated ovarian cancer [24]. Attacking tumour hypoxia via different mechanisms using NTG and a COX-2 inhibitor is an appealing prospect that is worthy of further investigation. Other options to target hypoxia in combination with NTG include mTOR inhibitors such as rapamycin [25] and temsirolimus [26], and/or metabolic agents such as metformin [27]. Additionally, there may also be an immunomodulatory aspect to celecoxib in that it may also be counter the possible MDSC promoting effects of NO by inhibiting production of MDSC [28–30].

### NO donation

The NO generating activity of NTG may also be of some value in cancer therapy:

- Statins – There is both epidemiological and pre-clinical evidence of an anti-cancer effect of lipophilic statins, including simvastatin, lovastatin and fluvastatin [31]. A number of clinical trials are also currently investigating the addition of statins to existing standard of care treatments in a range of cancers, including prostate, NSCLC, glioblastoma, colorectal and gastric cancers. Multiple mechanisms of action have been proposed for the anti-cancer effects of statins, and these are summarised in [31]. However, there are some indications that in breast cancer cells the cytotoxic effect of statins is related to NO generation [32, 33]. It is possible therefore that the cytotoxic effects of statins may be potentiated by the NO-donating activity of NTG. Given the low toxicity of statins and the potential activity against cancer, the combination with NTG and standard of care treatments warrants exploration in animal models to establish that this posited synergism does exist and can be exploited using standard dosing of these drugs.

### Immunomodulatory

There have been concerns regarding the possible effects of NO-donors on the immune system. Therefore it is of interest to investigate the effects of NTG with other agents to explore immunomodulatory activity. Pre-clinical evidence in this area is not sufficiently strong to warrant clinical trials and this may be a topic more suitable for initial exploration in animal models.

- Cimetidine – The classical H2 receptor antagonist (H2RA) cimetidine (Tagamet), primarily used to treat peptic ulcers and heartburn, has shown both in vitro, in vivo and clinical evidence of anti-cancer activity in a range of cancer types [34, 35]. Possessing a number of mechanisms of action, including cell adhesion, anti-angiogenesis and immunomodulation, there is especially strong evidence of a positive effect on survival when used peri-operatively for early stage colorectal cancer [36]. Given the concerns regarding the effect on cell mediated immunity of NTG, addressing this by targeting MDSC cells using cimetidine is an attractive prospect that warrants investigation in a clinical setting. In particular, the use of cimetidine and NTG in the peri-radiotherapy period would be an interesting avenue to explore with respect to maximising the potentially positive immune effects of radiotherapy [37].

### Other approaches

An alternative method of exploiting the EPR effect is to paradoxically induce systemic hypertension, (using angiotensin II, epinephrine or other anti-hypotensive) during the administration of chemotherapy. The increase in hypertension induces in an increase in tumour blood flow volume that results in greater accumulation of chemotherapeutic drug, whereas in non-tumour tissues the increase in hypertension induces an increased blood flow velocity. This effect has been shown in animal models [38, 39] and in patients [40, 41], resulting both in increased tumour regression and in sparing non-tumour tissues from toxicity. Whether it is possible to combine this approach with the application of NTG in some way to further maximise on the EPR effect is an intriguing question that has yet to be considered.

### Cancer stem cells

Finally, we speculate that NTG (independent of NO) may have an anti-cancer effect by inhibiting specific ALDH enzymes. It is known that NTG can cause deactivation of ALDH2; in fact long term clinical use of NTG gives rise to ‘nitrate tolerance’ where the drug no longer produces vasodilation. Withdrawal of the drug for a period of time restores its function. This phenomenon is attributed to the deactivation of the ALDH2 enzyme that at least partially catalyses the biotransformation of NTG. Wenzel *et al* showed that a single dose of NTG (0.8 mg) resulted in a 60% decrease in white blood cell ALDH2 activity [42]. Another study by Hink *et al* showed that in vivo treatment of cardiac bypass patients with NTG for more than 24 hours before surgery caused inhibition of ALDH2 [43]. These studies show that NTG administration results at least in intermittent inhibition of ALDH2.

The specificity of the Aldefluor assay, long assumed to be a specific inhibitor of ALDH1 has been opened to questioning and there is now some evidence that it may also inhibit other ALDH isoenzymes, including ALDH2 [44, 45]. This means that many studies which have assessed ALDH1 expression using only the Aldefluor assay may have not have been able to clearly differentiate between ALDH isoenzymes. This becomes significant in the context of the assessment of cancer cell stem populations using ALDH1 as a proxy marker. ALDH1 expression is associated with poor prognosis in breast [46], ovarian [47], esophageal [48] and other cancers.

In the case that ALDH2 is also a cancer stem cell marker then it is possible that inhibition of ALDH2 by clinically relevant doses of NTG may be a beneficial anticancer strategy.

## References

[1] Bashir A, Lewis MJ, Henderson AH (1982). Pharmacokinetic studies of various preparations of glyceryl trinitrate. Br J Clin Pharmaco.

[2] Jensen KM, Mikkelsen S (1997). Studies on the bioavailability of glyceryl trinitrate after sublingual administration of spray and tablet. Arzneimittel-Forsch.

[3] Curry SH, Aburawi SM (1984). Analysis, disposition and pharmacokinetics of nitroglycerin. Biopharm Drug Dispos.

[4] Weber S, de Lauture D, Rey E (1987). The effects of moderate sustained exercise on the pharmacokinetics of nitroglycerine. Br J Clin Pharmaco.

[5] Salvemini D, Pistelli A, Anggard E (1993). Vascular and anti-platelet actions of 1,2- and 1,3-glyceryl dinitrate. Br J Pharmaco.

[6] Burke AJ, Sullivan FJ, Giles FJ, Glynn SA (2013). The yin and yang of nitric oxide in cancer progression. Carcinogenesis.

[7] Choudhari SK, Chaudhary M, Bagde S (2013). Nitric oxide and cancer: a review. World J Surg Oncol.

[8] Fukumura D, Kashiwagi S, Jain RK (2006). The role of nitric oxide in tumour progression. Nat Rev Cancer.

[9] Burke JF, Mark VH, Soloway AH (1966). The effect of antibody to L-phenylalanine mustard conjugate on malignant cells selectively marked through ‘early inflammatory-like’ vascular permeability. Cancer Res.

[10] Matsumura Y, Maeda H (1986). A new concept for macromolecular therapeutics in cancer chemotherapy: mechanism of tumoritropic accumulation of proteins and the antitumor agent smancs. Cancer Res.

[11] Seymour LW, Ulbrich K, Steyger PS, Brereton M (1994). Tumour tropism and anti-cancer efficacy of polymer-based doxorubicin prodrugs in the treatment of subcutaneous murine B16F10 melanoma. Br J Cancer.

[12] Matthews NE, Adams MA, Maxwell LR (2001). Nitric oxide-mediated regulation of chemosensitivity in cancer cells. J Natl Cancer Inst.

[13] Postovit LM, Adams MA, Lash GE (2002). Oxygen-mediated regulation of tumor cell invasiveness Involvement of a nitric oxide signaling pathway. J Biol Chem.

[14] Postovit LM, Adams MA, Lash GE (2004). Nitric oxide-mediated regulation of hypoxia-induced B16F10 melanoma metastasis. Int J Cancer.

[15] Frederiksen LJ, Siemens DR, Heaton JP (2003). Hypoxia induced resistance to doxorubicin in prostate cancer cells is inhibited by low concentrations of glyceryl trinitrate. J Urol.

[16] Frederiksen LJ, Sullivan R, Maxwell LR (2007). Chemosensitization of cancer in vitro and in vivo by nitric oxide signaling. Clin Cancer Res.

[17] Muir CP, Adams MA, Graham CH (2006). Nitric oxide attenuates resistance to doxorubicin in three-dimensional aggregates of human breast carcinoma cells. Breast Cancer Res Treat.

[18] Siemens DR, Hu N, Sheikhi AK (2008). Hypoxia increases tumor cell shedding of MHC class I chain-related molecule: role of nitric oxide. Cancer Res.

[19] Barsoum IB, Hamilton TK, Li X (2011). Hypoxia induces escape from innate immunity in cancer cells via increased expression of ADAM10: role of nitric oxide. Cancer Res.

[20] Matsumura Y, Oda T, Maeda H (1987). [General mechanism of intratumor accumulation of macromolecules: advantage of macromolecular therapeutics]. Gan To Kagaku Ryoho.

[21] Wu J, Akaike T, Maeda H (1998). Modulation of enhanced vascular permeability in tumors by a bradykinin antagonist, a cyclooxygenase inhibitor, and a nitric oxide scavenger. Cancer Res.

[22] Tanaka S, Akaike T, Wu J (2003). Modulation of tumor-selective vascular blood flow and extravasation by the stable prostaglandin 12 analogue beraprost sodium. J Drug Target.

[23] Iyer AK, Greish K, Seki T (2007). Polymeric micelles of zinc protoporphyrin for tumor targeted delivery based on EPR effect and singlet oxygen generation. J Drug Target.

[24] Seki T, Fang J, Maeda H (2009). Enhanced delivery of macromolecular antitumor drugs to tumors by nitroglycerin application. Cancer Science.

[25] Nagai H, Yasuda H, Hatachi Y (2012). Nitric oxide (NO) enhances pemetrexed cytotoxicity via NO-cGMP signaling in lung adenocarcinoma cells in vitro and in vivo. Int J Oncol.

[26] Pantziarka P, Bouche G, Meheus L (2014). The repurposing drugs in oncology (ReDO) project. Ecancermedicalscience.

[27] Yasuda H, Yamaya M, Nakayama K (2006). Randomized phase II trial comparing nitroglycerin plus vinorelbine and cisplatin with vinorelbine and cisplatin alone in previously untreated stage IIIB/IV non-small-cell lung cancer. J Clin Oncol.

[28] Yasuda H, Nakayama K, Watanabe M (2006). Nitroglycerin treatment may enhance chemosensitivity to docetaxel and carboplatin in patients with lung adenocarcinoma. Clin Cancer Res.

[29] Yasuda H, Nakayama K, Sasaki T (2007). Partial response by nitroglycerin plus amrubicin regimen in patients with refractory and recurrent advanced non-small cell lung cancer who had received at least third-line chemotherapy. Cancer Ther.

[30] Reinmuth N, Meyer A, Hartwigsen D (2014). Randomized, double-blind phase II study to compare nitroglycerin plus oral vinorelbine plus cisplatin with oral vinorelbine plus cisplatin alone in patients with stage IIIB/IV non-small cell lung cancer (NSCLC). Lung Cancer.

[31] Dingemans A-MC (2014). A randomized phase II study of paclitaxel-carboplatin-bevacizumab (PCB) with or without nitroglycerin patches (NTG) in patients (pts) with stage IV nonsquamous non-small cell lung cancer (NSCLC): Nvalt 12 (NCT01171170). J Clin Oncol.

[32] Davidson A, Veillard AS, Tognela A (2014). 1245PA phase 3 randomised trial of adding nitroglycerin to first line chemotherapy for advanced non-small cell lung cancer: the Australasian lung cancer trials group nitro trial RANDOMISED TRIAL OF ADDING NITROGLYCERIN TO FIRST LINE CHEMOTHERAPY FOR ADVANCED NON-SMALL CELL LUNG CANCER: THE AUSTRALASIAN LUNG CANCER TRIALS GROUP NITRO TRIAL. Ann Oncol.

[33] Arrieta O, Blake M, de la Mata-Moya MD (2014). Phase II study Concurrent chemotherapy and radiotherapy with nitroglycerin in locally advanced non-small cell lung cancer. Radiother Oncol.

[34] Han JY, Nam BH, Kim HY (2012). A randomized phase II study of irinotecan plus cisplatin versus irinotecan plus capecitabine with or without isosorbide-5-mononitrate in advanced non-small-cell lung cancer. Ann Oncol.

[35] Siemens DR, Heaton JPW, Adams MA (2009). Phase II study of nitric oxide donor for men with increasing prostate-specific antigen-level after surgery or radiotherapy for prostate cancer. Urology.

[36] Liu Y, Chuang M, Tsai Y (2012). Nitroglycerine use in transcatheter arterial (chemo)embolization in patients with hepatocellular carcinoma and dual-energy CT assessment of Lipiodol retention. Eur Radiol.

[37] Illum H, Wang DH, Dowell JE (2015). Phase I dose escalation trial of nitroglycerin in addition to 5-fluorouracil and radiation therapy for neoadjuvant treatment of operable rectal cancer. Surgery.

[38] Chen Z, Zhang J, Stamler JS (2002). Identification of the enzymatic mechanism of nitroglycerin bioactivation. Proc Natl Acad Sci USA.

[39] Chen Z, Foster MW, Zhang J (2005). An essential role for mitochondrial aldehyde dehydrogenase in nitroglycerin bioactivation. Proc Natl Acad Sci USA.

[40] Mackenzie IS, Maki-Petaja KM, McEniery CM (2005). Aldehyde dehydrogenase 2 plays a role in the bioactivation of nitroglycerin in humans. Arterioscler Thromb Vasc Biol.

[41] Beretta M, Gruber K, Kollau A (2008). Bioactivation of nitroglycerin by purified mitochondrial and cytosolic aldehyde dehydrogenases. J Biol Chem.

[42] Tsou PS, Page NA, Lee SG (2011). Differential metabolism of organic nitrates by aldehyde dehydrogenase 1a1 and 2: substrate selectivity, enzyme inactivation, and active cysteine sites. AAPS J.

[43] Bonini MG, Stadler K, Silva SDO (2008). Constitutive nitric oxide synthase activation is a significant route for nitroglycerin-mediated vasodilation. Proc Natl Acad Sci USA.

[44] Mao M, Sudhahar V, Ansenberger-Fricano K (2012). Nitroglycerin drives endothelial nitric oxide synthase activation via the phosphatidylinositol 3-kinase/protein kinase B pathway. Free Radic Biol Med.

[45] Agvald P, Adding LC, Artlich A (2002). Mechanisms of nitric oxide generation from nitroglycerin and endogenous sources during hypoxia in vivo. Br J Pharmacol.

[46] Maeda H (2010). Nitroglycerin enhances vascular blood flow and drug delivery in hypoxic tumor tissues: analogy between angina pectoris and solid tumors and enhancement of the EPR effect. J Control Release.

[47] Jain RK (2013). Normalizing tumor microenvironment to treat cancer: bench to bedside to biomarkers. J Clin Oncol.

[48] Maeda H (2012). Vascular permeability in cancer and infection as related to macromolecular drug delivery, with emphasis on the EPR effect for tumor-selective drug targeting. Proc Jpn Acad Ser B Phys Biol Sci.

[49] Maeda H, Nakamura H, Fang J (2013). The EPR effect for macromolecular drug delivery to solid tumors: improvement of tumor uptake, lowering of systemic toxicity, and distinct tumor imaging in vivo. Adv Drug Deliv Rev.

[50] Wilson WR, Hay MP (2011). Targeting hypoxia in cancer therapy. Nat Rev Cancer.

[51] Ke Q, Costa M (2006). Hypoxia-inducible factor-1 (HIF-1). Mol Pharmacol.

[52] Sandau KB, Fandrey J, Brüne B (2001). Accumulation of HIF-1alpha under the influence of nitric oxide. Blood.

[53] Mateo J, García-Lecea M, Cadenas S (2003). Regulation of hypoxia-inducible factor-1alpha by nitric oxide through mitochondria-dependent and mitochondria-independent pathways. Biochem J.

[54] Sogawa K, Numayama-Tsuruta K, Ema M (1998). Inhibition of hypoxia-inducible factor 1 activity by nitric oxide donors in hypoxia. Proc Natl Acad Sci USA.

[55] Agani FH, Puchowicz M, Chavez JC (2002). Role of nitric oxide in the regulation of HIF-1alpha expression during hypoxia. Am J Physiol Cell Physiol.

[56] Cattaneo MG, Cappellini E, Benfante R (2011). Chronic deficiency of nitric oxide affects hypoxia inducible factor-1α (HIF-1α) stability and migration in human endothelial cells. PLoS ONE.

[57] Imtiyaz HZ, Simon MC (2010). Hypoxia-inducible factors as essential regulators of inflammation. Current topics in microbiology and immunology.

[58] Barsoum IB, Smallwood CA, Siemens DR (2014). A mechanism of hypoxia-mediated escape from adaptive immunity in cancer cells. Cancer Res.

[59] Bogdan C, Cuturi MC, Anegon I (2011). Regulation of Lymphocytes by Nitric Oxide. Methods in Molecular Biology.

[60] Bogdan C (2001). Nitric oxide and the immune response. Nat Immunol.

[61] Holán V, Krulová M, Zajícová A (2002). Nitric oxide as a regulatory and effector molecule in the immune system. Mol Immunol.

[62] Shoker AS, Yang H, Murabit MA (1997). Analysis of the in vitro effect of exogenous nitric oxide on human lymphocytes. Mol Cell Biochem.

[63] Krishnatry AS, Fung SM, Brazeau DA (2011). Nitroglycerin alters matrix remodeling proteins in THP-1 human macrophages and plasma metalloproteinase activity in rats. Nitric Oxide.

[64] Wesolowski R, Markowitz J, Carson WE (2013). Myeloid derived suppressor cells – a new therapeutic target in the treatment of cancer. J Immunother Cancer.

[65] Jayaraman P, Parikh F, Lopez-Rivera E (2012). Tumor-expressed inducible nitric oxide synthase controls induction of functional myeloid-derived suppressor cells through modulation of vascular endothelial growth factor release. J Immunol.

[66] Ushmorov A, Ratter F, Lehmann V (1999). Nitric-oxide-induced apoptosis in human leukemic lines requires mitochondrial lipid degradation and cytochrome C release. Blood.

[67] Millet A, Bettaieb A, Renaud F (2002). Influence of the nitric oxide donor glyceryl trinitrate on apoptotic pathways in human colon cancer cells. Gastroenterol.

[68] Leon-Bollotte L, Subramaniam S, Cauvard O (2011). S-nitrosylation of the death receptor fas promotes fas ligand-mediated apoptosis in cancer cells. Gastroenterol.

[69] Van der Veldt AAM, Lubberink M, Bahce I (2012). Rapid decrease in delivery of chemotherapy to tumors after anti-VEGF therapy: implications for scheduling of anti-angiogenic drugs. Cancer Cell.

[70] Turley RS, Fontanella AN, Padussis JC (2012). Bevacizumab-induced alterations in vascular permeability and drug delivery: a novel approach to augment regional chemotherapy for in-transit melanoma. Clinical Cancer Res.

[71] Dickson PV, Hamner JB, Sims TL (2007). Bevacizumab-induced transient remodeling of the vasculature in neuroblastoma xenografts results in improved delivery and efficacy of systemically administered chemotherapy. Clin Cancer Res.

[72] Arjaans M, Oude Munnink TH, Oosting SF (2013). Bevacizumab-induced normalization of blood vessels in tumors hampers antibody uptake. Cancer Res.

[73] Selvakumaran M, Yao KS, Feldman MD (2008). Antitumor effect of the angiogenesis inhibitor bevacizumab is dependent on susceptibility of tumors to hypoxia-induced apoptosis. Biochem Pharmacol.

[74] Miyazaki S, Kikuchi H, Iino I (2014). Anti-VEGF antibody therapy induces tumor hypoxia and stanniocalcin 2 expression and potentiates growth of human colon cancer xenografts. Int J Cancer.

[75] Sakata S, Yoshihara T, Arima H (2011). Differential effects of organic nitrates on arterial diameter among healthy Japanese participants with different mitochondrial aldehyde dehydrogenase 2 genotypes: randomised crossover trial. BMJ open.

[76] Reyman B, Van Gisbergen M, Zegers K (2015). Nitroglycerin as a sensitizer in the treatment of non small cell lung cancer: from cells in vitro to phase 3 trial. Radiother Oncol.

[77] Taurin S, Nehoff H, Greish K (2012). Anticancer nanomedicine and tumor vascular permeability; where is the missing link?. J Control Release.

[78] Nichols JW, Bae YH (2014). EPR: evidence and fallacy. J Control Release.

[79] Jain RK (1990). Vascular and interstitial barriers to delivery of therapeutic agents in tumors. Cancer Metastasis Rev.

[80] Stohrer M, Boucher Y, Stangassinger M (2000). Oncotic pressure in solid tumors is elevated. Cancer Res.

